# First person – Brandon Hastings

**DOI:** 10.1242/bio.060176

**Published:** 2023-11-10

## Abstract

First Person is a series of interviews with the first authors of a selection of papers published in Biology Open, helping researchers promote themselves alongside their papers. Brandon Hastings is first author on ‘ Melanistic coloration does not influence thermoregulation in the crepuscular gecko *Eublepharis macularius*’, published in BiO. Brandon is a data analyst in the lab of Ylenia Chiari at Fairfax, USA, investigating disease evolution and comparative genetics in vertebrate species, specifically focusing on topics that could potentially improve our understanding of human diseases.


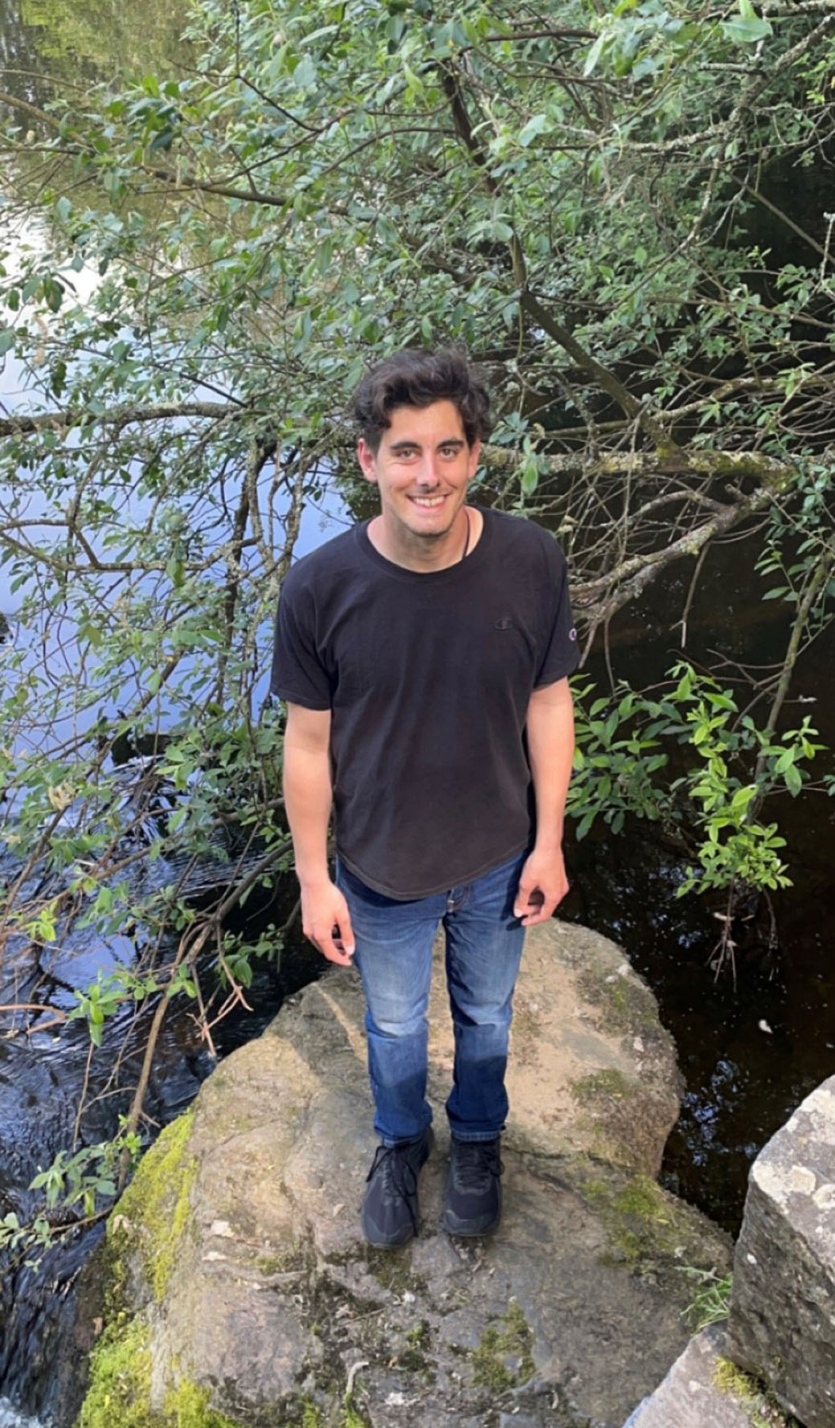


**Brandon Hastings**

**Describe your scientific journey and your current research focus**

My scientific journey feels a lot like going around trying all the free samples in an ice cream shop. I started off doing undergrad research on bird and insect biomechanics, I had a short-lived thesis proposal on floral coloration evolution, and my final thesis focused on investigating reptile coloration and thermoregulation. Then I took a break to become a data analyst and enjoy a working holiday visa in New Zealand for the next year. To top it all off, I plan to get my PhD investigating cancer or disease evolution in vertebrates, with potential ramifications for human health. Thanks to these experiences, I've built a diverse set of skills that I think can be applied to some interesting projects in the future.

My scientific journey feels a lot like going around trying all the free samples in an ice cream shop.

**Who or what inspired you to become a scientist?**

Truthfully, what originally inspired me to become a scientist was reaching the end of my pre-medical education and realizing the high cost of medical school and my newfound fear of human medical procedures. I made the right choice though because science allows me to constantly keep learning, and that is probably one of my favourite things.

**What are the potential implications of this finding for your field of research?**

Our data confirms previous studies that suggested that the crepuscular gecko *E. macularius* absorbs heat from its surroundings to maintain its body temperature. Furthermore, previous studies on other organisms, including reptiles, suggest that melanistic coloration can be used to help in maintaining and regulating the internal body temperature of the animal. However, these data mostly come from diurnal animals and we currently do not know the role of melanistic coloration for thermoregulation in animals that are active in dim light. In our work, we found that the melanistic colour and colour pattern of *E. macularius* does not seem to be involved in thermoregulation. As such, melanistic coloration in this species may have other functions. As with most research projects, our project produced results that will serve as a baseline to further investigate the function of body coloration in this and other crepuscular and nocturnal species.

**Figure:**
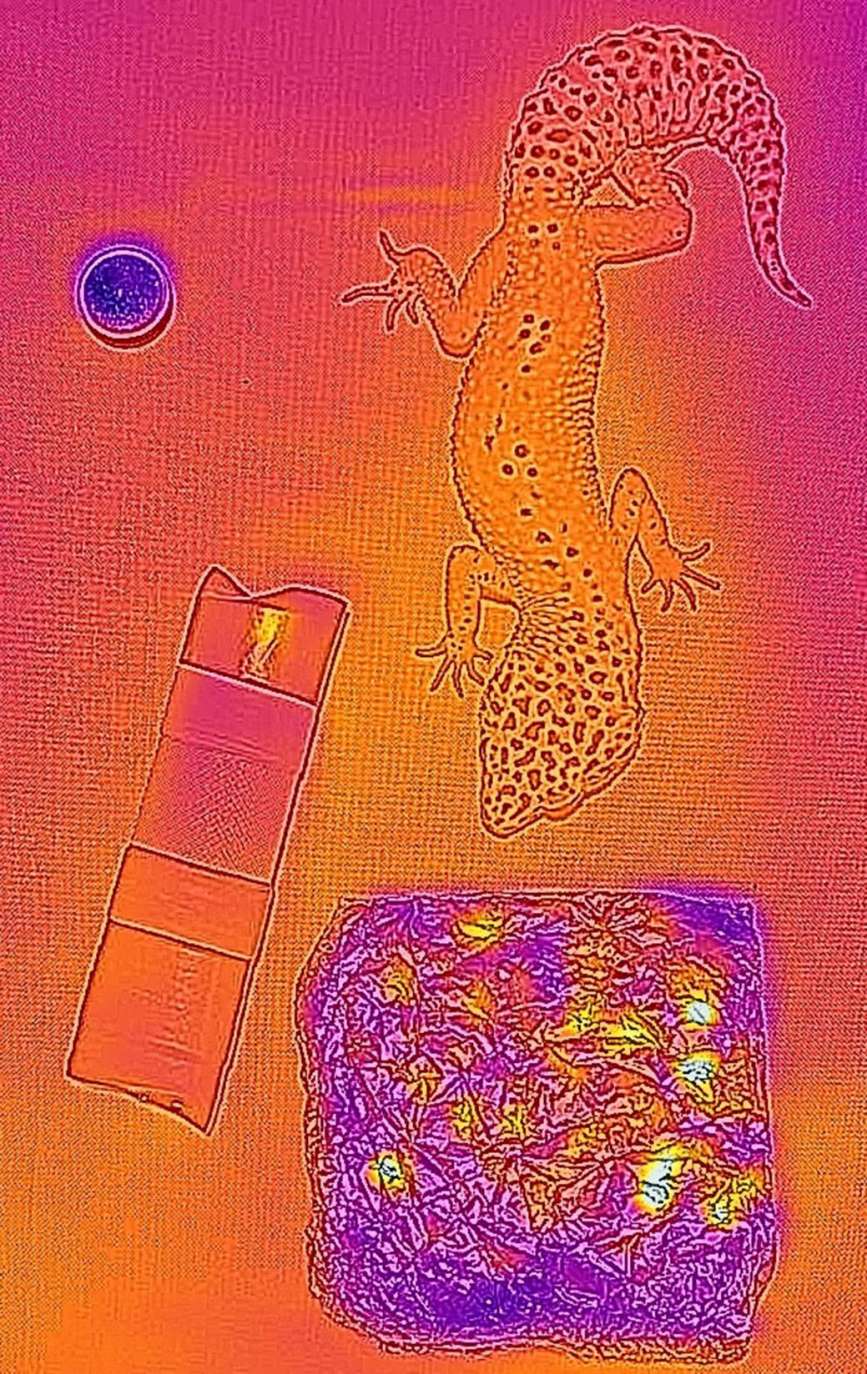
Thermal image of a gecko in the experimental terrarium.

**Which part of this research project was the most rewarding?**

Being able to collaborate with so many people that helped us implement some very interesting technology into this project was wonderful. Our co-authors from CIBIO in Portugal are amazing with teaching us to use thermal imaging for reptile temperature measurements. We were also able to collaborate with researchers at George Mason University who use computer vision to tackle infrastructural engineering problems, so having them adapt that knowledge to study a biological system was very exciting. I'm a big proponent of blending fields to answer biological questions, so bringing everyone together for this project was important to me.

**What do you enjoy most about being an early-career researcher?**

My favourite part of being an early-career researcher is coming to recognize all of the skills that I have built up both during my master's degree and while drafting this paper that will make me a better researcher in the future. I believe imposter syndrome is very prevalent in this line of work, and being able to look back on that time that was spent wondering if I'm good enough to be in this field and recognize these small incremental improvements to how you operate as a scientist is a very rewarding experience.

**What piece of advice would you give to the next generation of researchers?**

Above all, find research that makes you feel fulfilled at the end of the day – regardless of the perceived impact – and find colleagues that share that feeling.

**What's next for you?**

Up next is relaxing and traveling in New Zealand while preparing to start a PhD program next year.
